# Early Enteral Nutrition May Improve Survival in Patients With Cardiogenic Shock

**DOI:** 10.1155/emmi/1465194

**Published:** 2025-01-06

**Authors:** Liangliang Zheng, Jingwei Duan, Baomin Duan

**Affiliations:** ^1^Emergency Department, Beijing Hospital, National Center of Gerontology, Institute of Geriatric Medicine, Chinese Academy of Medical Sciences, Beijing, China; ^2^Emergency Department, Peking University Third Hospital, Beijing, China; ^3^Emergency Department, Kaifeng Central Hospital, Kaifeng, China

**Keywords:** cardiogenic shock, clinical outcomes, early enteral nutrition, MIMIC-IV, propensity score-matched analysis

## Abstract

**Background and Aim:** International guidelines recommend early enteral nutrition (EEN) for critically ill patients. However, evidence supporting the optimal timing of EN in patients diagnosed with cardiogenic shock (CS) is lacking. As such, this study aimed to compare the clinical outcomes and safety of EEN versus delayed EN in patients diagnosed with CS.

**Methods:** This retrospective cohort study was conducted using data from the Medical Information Mart for Intensive Care IV version 2.2 database. Patients who received EN within 2 days of admission were assigned to the EEN group. A 1:1 propensity score-matched (PSM) analysis was performed to control for bias in baseline characteristics and ensure the reliability of the results. To exclude the impact of confounders, an adjusted proportional hazards regression model was used to verify the independence between EEN and survival outcomes.

**Results:** Of 1846 potentially eligible patients, 1398 received EEN and 448 received delayed EN. After 1:1 PSM, 818 patients were assigned to the EEN (*n* = 409) and delayed EN (*n* = 409) groups. Regarding cumulative survival, patients with CS receiving EEN experienced better 30-, 90-, and 180-day survival outcomes than the delayed EN group (hazard ratio [HR] 0.803 [95% confidence interval [CI] 0.647–0.998], *p*=0.045; HR 0.729 [95% CI 0.599–0.889], *p*=0.001; and HR 0.778 [95% CI 0.644–0.938], *p*=0.008, respectively). After adjusting for confounders, EEN was found to be independently associated with survival outcomes. Moreover, EEN did not increase the risk(s) for ileus, aspiration pneumonia, or gastrointestinal bleeding. Patients who received delayed EN experienced longer hospital stays than those receiving EEN (17 days [interquartile range [IQR] 10–25] versus 12 days [IQR 7–19 days], respectively; *p* < 0.001).

**Conclusion:** EEN was not associated with harm, but rather with improved survival outcomes in patients diagnosed with CS. Further studies are required to verify these findings.

## 1. Introduction

Cardiogenic shock (CS) is primarily caused by acute myocardial infarction, chronic heart failure, and/or structural heart disease [1]. Due to the increasing incidence of these diseases, the number of patients diagnosed with CS is also increasing [2]. Treatment of early CS is beginning to be emphasized; however, once patients progress to the clinical stage of CS, the short-term mortality rate remains as high as 40%–60% [3]. When patients reach the clinical stage of CS, clinicians devote more attention to maintaining blood pressure or improving cardiac function in the intensive care unit (ICU) or cardiac care unit (CCU); however, in doing so, nutritional support may be neglected.

Metabolic levels are significantly higher in critically ill patients [4], which results in higher caloric demands to support this increased metabolism [5]. Inadequate nutritional support may be associated with various adverse events including bedsores and infections in the ICU [6]. In addition, previous studies and experience have shown that early enteral nutrition (EEN) may be contraindicated in patients diagnosed with circulatory shock [7–9]. A previous study reported that, in patients with circulatory shock, EEN (< 48 h) and delayed EN (≥ 48 h) yielded no apparent difference in short-term survival and other clinical outcomes [10]. However, patients receiving EEN may have a greater risk for digestive complications [11]. Although parenteral nutrition (PN) can provide a similar caloric supply, the fluid load from PN can be extremely detrimental to patients diagnosed with CS [12]. This fluid load could increase cardiac preload and further worsen cardiac function, leading to the exacerbation of CS [13]. Due to the unique pathophysiology of CS, these findings and experiences in other shock settings are not applicable to patients with CS; therefore, extensive or total PN may not be appropriate in this patient population.

Providing adequate nutritional support in patients diagnosed with CS is an urgent task for clinicians. Accordingly, this study compared clinical outcomes among patients receiving EEN versus delayed EN, and aimed to explore the most appropriate strategy for nutritional support for those diagnosed with CS.

## 2. Materials and Methods

### 2.1. Study Design

This retrospective cohort study used data from the Medical Information Mart for Intensive Care IV (MIMIC-IV), a large electronic database. Information in the MIMIC-IV database is contained in four parts: emergency department, admissions, ICU, and follow-up. All data used in the present study were extracted from the most recent version of the MIMIC-IV (version 2.2), released in January 2023, which contains data from > 400,000 patients admitted to the ICU at a single center (Beth Israel Deaconess Medical Center, Boston, MA, USA) from 2008 to 2019. Importantly, the data contained in the MIMIC-IV is de-identified. Patient identifiers were removed according to the Health Insurance Portability and Accountability Act Safe Harbor provisions, and a reprogrammed system number was used to identify and access each patient's records from this database. The system includes a “subject_id” for every patient, a “hadm_id” for every hospital admission, and a “stay_id” for every ICU admission. For example, a patient who has been hospitalized 3 times and admitted to the ICU once will have 1 subject_id, 3 hadm_id, and 1 stay_id [14]. Before accessing this database, the CITI Data or Specimens Only Research certification was required (the authors' certification can be verified at <https://www.citiprogram.org/verify/?k026a8801-1124-40bb-ac72-c64d66bdab42-36003834>; the authors also needed to agree to the PhysioNet Credentialed Health Data Use Agreement 1.5.0, which contains a related ethics statement (https://www.physionet.org/content/mimiciv/view-dua/2.2/) [15]. Therefore, this study did not require any additional ethics approval.

### 2.2. Inclusion and Exclusion Criteria

The inclusion criteria were as follows: diagnosed with CS (International Classification of Diseases, Ninth or 10th Revision [ICD-9 code 78551 and ICD-10 code R570]); age ≥ 18 years; and any cause(s) of CS. The exclusion criteria were as follows: pregnancy; received only PN during hospitalization; intestinal obstruction, GIB, and other contraindications to EN; underwent any abdominal procedure or any other examination or procedure needed to pause EN for 48 h; any malignant tumor and life expectancy < 1 year; moribund patients who died within 48 h of admission; and incomplete/unavailable information.

### 2.3. Data Extraction

Emergency department data extracted included vital signs and arterial blood gases measured on admission. Admissions data extracted included age, sex, previous disease(s), treatment during the first 2 days (percutaneous coronary intervention, extracorporeal membrane oxygenation and therapeutic hypothermia, pacemaker), and causes of CS. In the ICU, another therapy during the first 2 days (continuous renal replacement therapy [CRRT], intra-aortic balloon pump [IABP], ventricular-assist device [i.e., Impella], left atrial-to-femoral artery bypass system [e.g., TandemHeart] and ventilator), medications during the first 2 days (vasoactive-inotropic score [VIS] calculated based on medication[s] dosage), and the first sequential organ failure assessment (SOFA) score in the ICU or CCU were extracted. During follow-up, cumulative survival (in days) and safety endpoints (ileus, aspiration pneumonia, and GIB) were determined. Data from the above variables were extracted using ICD-9 or ICD-10 codes, and hadm_id, or stay_id, which is a unique number for each variable or patient.

### 2.4. Endpoint Definitions

The primary endpoint was defined as 30-day survival. Safety endpoints included ileus, aspiration pneumonia, and GIB. Secondary endpoints were defined as survival at 90 and 180 days. EEN was defined as EN ≤ 48 h after admission, and delayed EN was defined as EN > 48 h after admission.

### 2.5. Statistical Analysis

Non-normally distributed continuous variables are expressed as median (interquartile range [IQR]), while normally distributed variables are expressed as mean (standard deviation [SD]). Categorical variables are expressed as total number and percentage. The Student's *t*-test was used to compare normally distributed continuous variables, and the Mann–Whitney *U* test was used for data with a non-normal distribution. The *χ*^2^ test or Fisher's exact test were used to compare categorical variables. Differences with *p*  <  0.05 were considered to be statistically significant.

A 1:1 propensity score-matched (PSM) analysis was performed to balance possible confounders between EEN and delayed EN. The standard deviation of the logit of the propensity score was set at 0.02. Cases with higher propensity scores were matched first. To better eliminate the result bias caused by confounding factors, all variables except clinical outcomes (i.e., mortality at 30, 90, and 180 days) and safety endpoints (ileus, aspiration pneumonia, and GIB) were included in PSM.

Kaplan–Meier and Cox-proportional hazard models were used to calculate hazard ratio (HR) with corresponding 95% confidence interval (CI), with *p*  <  0.05 considered to be statistically significant. Adjusted proportional hazards (Cox) regression models were used to verify the independence of the association between EN initiation and survival outcomes to control for prehospital characteristics (age, sex, body mass index [BMI], and all included comorbidities) and hospitalization characteristics (cause of CS, vital signs, arterial blood gas, medications, therapy, VIS, and SOFA score). Furthermore, sensitivity analysis was performed to compare differences in the primary endpoint in patients with specific conditions.

All statistical analyses were performed using SPSS version 25.0 (IBM Corp., Armonk, NY, USA) for Windows (Microsoft Corp., Redmond, WA, USA).

## 3. Results

**Figure 1:**
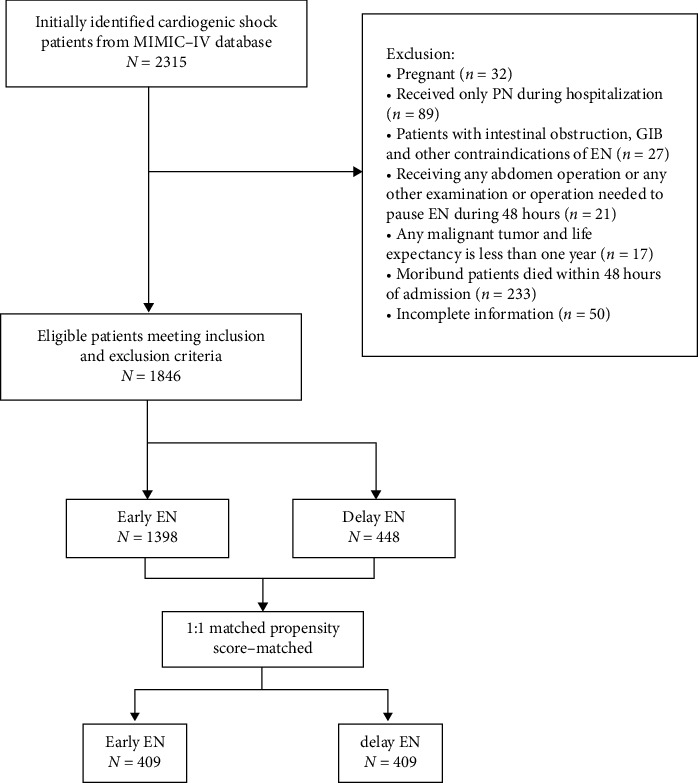
Flowchart. EN, enteral nutrition; GIB, gastrointestinal bleeding; PN, parenteral nutrition.

Of 2315 patients initially identified, 1846 were eligible for inclusion in the study, of whom 1398 (76%) were treated with EEN and 448 (24%) were treated with delayed EN (Figure 1).

### 3.1. Baseline Characteristics

The crude baseline characteristics revealed that the median age of the patients was 68 years (IQR 58–78 years) and 1133 (61%) were male. The median BMI was 28.1 kg/m^2^ (IQR 24.0–32.7 kg/m^2^). More patients in the EEN group experienced previous myocardial infarction than in the delayed EN group (52% versus [vs.] 43%, respectively; *p*=0.001). However, more patients in the delayed EN group had previous diabetes mellitus, renal dysfunction and stroke those in the EEN group (38% vs. 43% [*p*=0.031]; 38% vs. 47% [*p* < 0.001]; 11% vs. 14% [*p*=0.034], respectively). The causes of CS differed significantly between the 2 groups. The mean arterial pressure at admission for delayed EN group is lower than that for EEN group (56 mmHg [49–63] vs. 54 mmHg [46–60]; *p* < 0.001). Regarding treatment during the first 2 days, there were statistical differences between the 2 groups among patients receiving CRRT and IABP. Patients in the delayed EN group may have experienced more severe symptoms than those in the EEN group because the SOFA score was higher in the delayed EN group (7 [IQR 4–11] vs. 7 [IQR 5–11]; *p*=0.037). Patients in the delayed EN group experienced longer hospital stays, although ICU stays did not differ significantly between the 2 groups.

Regarding clinical outcomes and complications, the 30-, 90-, and 180-day mortality rates were higher in the delayed EN group than those in the EEN group (40% vs. 32% [*p*=0.004]; 50% vs. 40% [*p* < 0.001]; 55% vs. 45% [*p* < 0.001], respectively). The incidence of aspiration pneumonia was higher in the delayed EN group than that in the EEN group (7% vs. 4%, respectively; *p*=0.020). The crude baseline characteristics are summarized in Table 1.

### 3.2. PSM Analysis

After PSM, 818 patients were included in this study. The 818 patients were divided equally into the EEN and delayed EN groups. The median age of the patients was 68 years (IQR 58–77) and 483/919 (59%) were male. The median BMI of the overall cohort was 28.3 kg/m^2^ (IQR 24.1–33.4 kg/m^2^). Moreover, there were no statistical differences in baseline characteristics between the 2 groups (Table 2). Although ICU stays were similar between the 2 groups (*p*=0.584), median hospital stays in the delayed EN group were significantly longer than those in the EEN group (17 days [IQR 10–25 days] vs. 12 days [IQR 7–19 days]; *p* < 0.001).

Regarding cumulative survival at 30, 90, and 180 days, analysis revealed improved survival outcomes in the EEN group compared with the delayed EN group (HR 0.803 [95% CI 0.647–0.998], *p*=0.045; HR 0.729 [95% CI 0.599–0.889], *p*=0.001; HR 0.778 [95% CI 0.644–0.938], *p*=0.008, respectively). After adjusting for prehospital and/or hospitalization characteristics, EEN was found to be independently associated with improved survival outcomes (Supporting Table 1).

**Figure 2:**
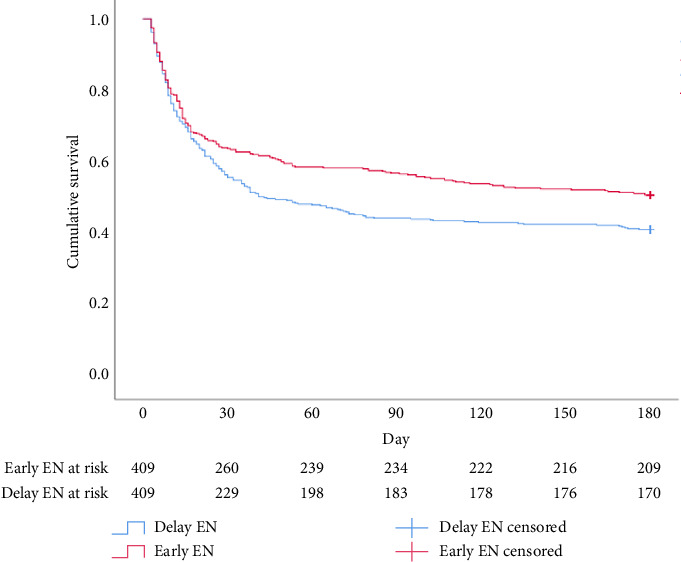
Kaplan–Meier survival curve for 180-day survival.

**Figure 3:**
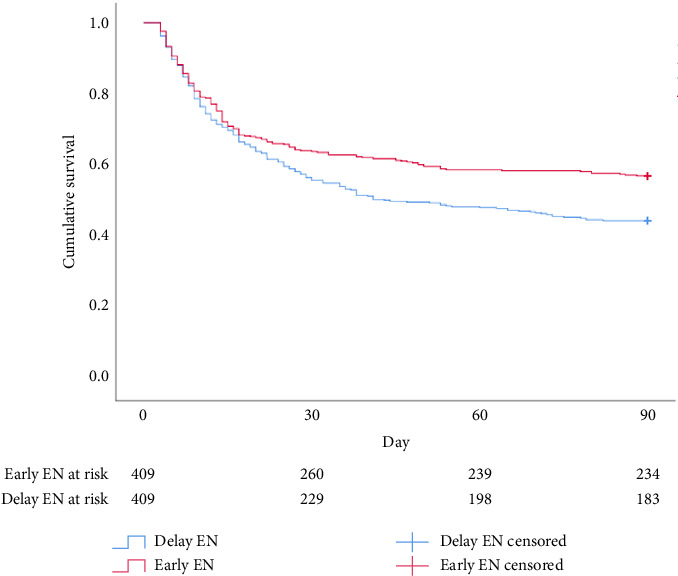
Kaplan–Meier survival curve for 90-day survival.

**Figure 4:**
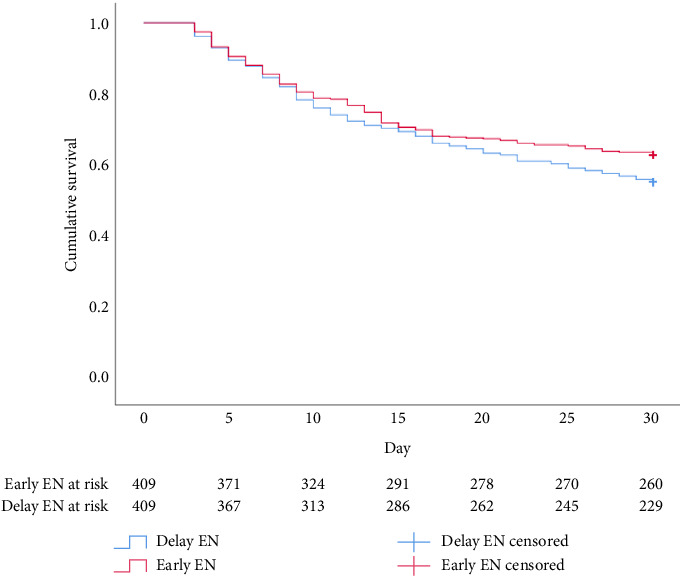
Kaplan–Meier survival curve for 30-day survival.

Regarding clinical outcomes, consistent results in mortality between the 2 groups were revealed at 30 days (36% vs. 44%, odds ratio [OR] 0.729 [95% CI 0.551–0.965]; *p*=0.016), 90 days (43% vs. 55%, OR 0.606 [95% CI 0.459–0.798]; *p* < 0.001) and 180 days (49% vs. 58%, OR 0.681 [95% CI 0.516–0.897]; *p*=0.004) (Figures 2, 3, and 4). Furthermore, compared with delayed EN, EEN did not significantly increase the occurrence of complications such as ileus, aspiration pneumonia, and GIB (Table 3).

### 3.3. Sensitivity Analysis

**Figure 5:**
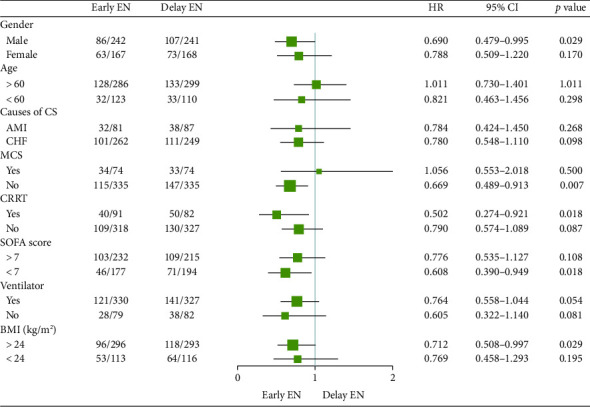
Sensitive analysis of primary outcome. (BMI, body mass index; CRRT, continuous renal replacement therapy; CS, cardiogenic shock; EN, enteral nutrition; MCS, mechanical circulatory support; SOFA, sequential organ failure assessment).

Sensitivity analysis revealed that males may benefit more from EEN than from delayed EN (OR 0.690 [95% CI 0.479–0.995]; *p*=0.029). Patients receiving mechanical circulatory support may have better survival outcomes after EEN. Patients with mild CS and SOFA score < 7 also exhibited a higher survival rate in the EEN group. Finally, a better survival outcome was observed for patients in the EEN group with a BMI > 24 kg/m^2^ (Figure 5).

## 4. Discussion

In this retrospective cohort study, EEN was associated with improved survival outcomes. However, an earlier report suggested that EN is associated with an increase in mesenteric arterial output and that EN could be detrimental by overwhelming the mechanisms of mesenteric adaptation. Moreover, EN may increase the risk for mesenteric ischemia, bacterial translocation, and sepsis in patients diagnosed with CS. Thus, early guidelines indicated that EN should be prudently used within 72 h of CS [16].

As research continues, there has been a significant shift in attitudes toward EEN in patients with shock. The multicenter, randomized controlled trial (RCT) NUTRIREA-2 revealed that, compared with early PN, EEN did not increase the risk for mortality (35% vs. 37%; *p*=0.33) or the occurrence of secondary infection (14% vs. 16%; *p*=0.25) [11]. Similarly, another multicenter RCT revealed no statistical difference in 30-day mortality between the EEN and PN groups in patients requiring ICU admission [17]. Accordingly, guidelines have changed in recent years and recommend the use of EEN in the majority of critically ill patients, although with specific precautions [18]. Moreover, for patients with circulatory shock undergoing mechanical ventilation, EEN may be associated with improved clinical outcomes and more ICU-free days [10]. The above evidence suggests that EN is a suitable method for calorie support and EEN may be more suitable for patients in the ICU. However, this evidence from circulatory shock cannot be directly applied to patients with CS. Due to the lack of evidence on CS, our clinical decisions must draw from this evidence, which does not appear appropriate. To date, only 1 one retrospective study has compared the clinical outcomes of EEN versus delayed EN in patients with CS [19]. That study reported that, compared with delayed EN, EEN could lower mortality (HR 0.78 [95% CI 0.62–0.98]; *p*=0.03) for patients with CS or obstructive shock requiring extracorporeal membrane oxygenation. Therefore, we believe that EEN (within 2 days) is a reasonable feeding strategy for patients with CS.

However, the unique pathophysiological features of CS are completely different from those of other shock types. Rapid deterioration of cardiac function due to primary or secondary cardiac disease is the pathological basis for the development of CS [20]. Therefore, volume management in patients with CS is important [21]. Excessive fluid load can further deteriorate cardiac function, making it more difficult to correct circulatory failure. This is different from other types of shock, such as septic or anaphylactic shock, which require massive fluid replacement to maintain an effective circulating blood volume and improve circulatory collapse [22, 23]. Therefore, the fluid load associated with PN may be unacceptably high in patients with CS. In addition, because the caloric density of EN is much higher than that of PN, EN can provide far more calories than PN in the same volume [24]. Fluid load restriction in patients with CS using PN often results in an inability to consume sufficient calories; as such, EN has clear advantages in this regard. The mesenteric arteries are diastolic and require a large blood supply after receiving EN [25], which is a “double-edge” sword in patients with CS. Previous studies have shown that mesenteric artery diastole exacerbates the volume distribution imbalance in patients with shock receiving EN, further worsening circulatory collapse [26, 27]. Moreover, incomplete mesenteric artery diastole in this state makes it more likely to cause gastrointestinal ischemia, which could lead to diarrhea, intestinal obstruction, and even systemic infections due to the displacement of the intestinal flora [28–30]. However, in CS, diastole of the mesenteric artery could potentially reduce cardiac afterload. Previous studies have shown that reducing afterload in patients with CS is effective in improving prognostic outcomes [31, 32]. Furthermore, EEN lowers the risk for infectious complications. EEN can help maintain the integrity of the intestinal mucosal barrier and reduce bacterial translocation from the small intestine [33]. The integral gut mucosa not only lowers bacterial translocation but also decreases toxin levels, oxidative stress, and inflammatory factor release, maintaining the gut barrier [34]. However, inflammatory factors, such as interleukin-6 and interleukin-18, can further exacerbate CS [35, 36]. Therefore, this may be the pathophysiological basis by which EEN improves clinical outcomes and reduces complications in patients with CS.

Sensitivity analysis revealed that males with a SOFA score < 7 and without mechanical circulatory support receiving EEN may experience better clinical outcomes than those receiving delayed EN. We hypothesized that these 2 patient groups were less severely ill and, therefore, better tolerated EN. Interestingly, a recent study reported that patients with CS after cardiac surgery and those intolerant to EEN had a worse prognosis [37].

These potential mechanisms may be associated with EEN and the improved clinical outcomes in patients with CS. Carefully designed RCTs are required to verify the findings of the present study.

### 4.1. Limitations

Despite our best attempts to mitigate potential bias, the present study had some limitations, the first of which was its single-center retrospective cohort design. As such, we could not avoid the overall diversity of patients included. Second, due to data limitations in the database, it was not possible to extract data regarding calorie intake. Finally, due to the lack of previous randomization, the subjective decisions of clinicians may have potentially influenced the interventions and outcomes.

## 5. Conclusions

Based on the results of this study, EEN was not associated with harm but rather with improved survival in patients with CS. Therefore, EEN may be a reasonable feeding strategy for patients with CS. Moreover, patients with CS, who are less ill and do not undergo mechanical circulatory support, are more likely to benefit from EEN. A multicenter RCT is required to verify this finding.

## Figures and Tables

**Table 1:** Baseline characteristics for all patients.

	All patients *N* = 1846	Early EN *N* = 1398	Delay EN *N* = 448	*p* value
Age (year), median (IQR)	68 (58–78)	69 (59–78)	68 (58–77)	0.631
Male, *n* (%)	1133 (61)	869 (62)	264 (60)	0.122
BMI (kg/m^2^), median (IQR)	28.1 (24.0–32.7)	28.1 (24.1–32.7)	28.1 (23.8–32.6)	0.812
Smoking, *n* (%)	582 (32)	421 (30)	161 (36)	0.013
Previous diseases				
Myocardial infarction, *n* (%)	914 (50)	721 (52)	193 (43)	0.001
Heart failure, *n* (%)	1513 (82)	1139 (81)	374 (83)	0.187
Diabetes mellitus, *n* (%)	728 (39)	534 (38)	194 (43)	0.031
Renal dysfunction, *n* (%)	746 (40)	534 (38)	212 (47)	< 0.001
^#^Stroke, *n* (%)	224 (12)	158 (11)	66 (14)	0.034
Causes of CS				0.001
Acute myocardial infarction, *n* (%)	523 (28)	428 (31)	95 (21)	
Congestive heart failure, *n* (%)	1035 (56)	766 (55)	269 (60)	
Other, *n* (%)	288 (16)	204 (15)	84 (19)	
Vital sign at admission				
Heart rate, median (IQR)	106 (92–123)	106 (92–123)	107 (93–123)	0.603
Mean arterial pression, median (IQR)	55 (48–62)	56 (49–63)	54 (46–60)	< 0.001
Arterial blood gas at admission				
pH, median (IQR)	7.31 (7.22–7.38)	7.31 (7.22–7.37)	7.32 (7.22–7.39)	0.146
Lactate (mmol/L), median (IQR)	2.5 (1.5–4.6)	2.4 (1.5–4.4)	2.8 (1.5–5.5)	0.080
Therapy during first 2 days				
PCI, *n* (%)	309 (17)	240 (17)	70 (16)	0.247
CRRT, *n* (%)	298 (16)	207 (15)	91 (20)	0.004
IABP, *n* (%)	319 (17)	264 (19)	55 (12)	0.001
ECMO, *n* (%)	29 (2)	19 (1)	10 (2)	0.142
Impella, *n* (%)	75 (4)	55 (4)	20 (4)	0.354
TandemHeart, *n* (%)	24 (1)	19 (1)	5 (1)	0.454
Ventilator, *n* (%)	1360 (74)	1017 (73)	343 (77)	0.062
Therapeutic hypothermia, *n* (%)	15 (1)	11 (1)	4 (1)	0.513
Pacemaker, *n* (%)	137 (7)	104 (7)	33 (7)	0.527
Medications during first 2 days				
Epinephrine, *n* (%)	374 (20)	262 (19)	112 (25)	0.003
Norepinephrine, *n* (%)	801 (43)	623 (45)	178 (40)	0.041
Phenylephrine, *n* (%)	487 (26)	356 (25)	131 (29)	0.065
Vasopressin, *n* (%)	510 (28)	362 (26)	148 (33)	0.002
Dopamine, *n* (%)	413 (22)	315 (23)	98 (22)	0.413
Dobutamine, *n* (%)	509 (28)	389 (28)	120 (27)	0.358
Muscle relaxant, *n* (%)	171 (9)	121 (9)	50 (11)	0.069
⁣^∗^Max VIS, median (IQR)	19 (7–31)	17 (6–28)	23 (10–36)	< 0.001
SOFA score at admission, median (IQR)	7 (4–11)	7 (4–11)	7 (5–11)	0.037
Hospital stays (day), median (IQR)	12 (7–20)	10 (6–17)	17 (10–25)	< 0.001
ICU stays (day), median (IQR)	6 (4–11)	5 (3–10)	6 (4–11)	0.071
Clinical outcomes				
Mortality at 30 days	627 (35)	451 (32)	176 (40)	0.004
Mortality at 90 days	786 (47)	556 (40)	230 (51)	< 0.001
Mortality at 180 days	871 (47)	626 (45)	245 (55)	< 0.001
Complications				
Ileus, *n* (%)	37 (2)	28 (2)	9 (2)	0.561
Aspiration pneumonia, *n* (%)	91 (5)	61 (4)	31 (7)	0.020
GIB, *n* (%)	117 (6)	77 (6)	40 (9)	0.008

Abbreviations: BMI, body mass index; CRRT, continuous renal replacement therapy; ECMO, extracorporeal membrane oxygenation; EN, enteral nutrition; GIB, gastrointestinal bleeding; IABP, intra-aortic balloon pump; ICU, intensive care unit; IQR, interquartile range; PCI, percutaneous coronary intervention; SOFA, sequential organ failure assessment; VIS, vasoactive-inotropic score.

^#^: stroke include both of ischemic and hemorrhagic.

⁣^∗^MAX VIS is the maximum within 48 h of admission.

**Table 2:** Baseline characteristics after propensity score matching.

	All patients *N* = 818	Early EN *N* = 409	Delay EN *N* = 409	*p* value
Age (year), median (IQR)	68 (58–77)	69 (58–77)	68 (58–77)	0.568
Male, *n* (%)	483 (59)	242 (59)	241 (59)	0.500
BMI (kg/m^2^), median (IQR)	28.3 (24.1–33.0)	28.3 (24.2–33.4)	28.2 (24.0–32.6)	0.629
Smoking, *n* (%)	282 (34)	142 (35)	140 (34)	0.471
Previous diseases				
Myocardial infarction, *n* (%)	345 (42)	170 (42)	175 (43)	0.389
Heart failure, *n* (%)	668 (82)	329 (80)	339 (83)	0.208
Diabetes mellitus, *n* (%)	350 (43)	175 (43)	175 (43)	0.528
Renal dysfunction, *n* (%)	373 (46)	185 (45)	188 (46)	0.444
Stroke^#^, *n* (%)	131 (16)	71 (17)	60 (15)	0.170
Causes of CS				0.329
Acute myocardial infarction, *n* (%)	209 (26)	99 (24)	110 (27)	
Congestive heart failure, *n* (%)	471 (58)	245 (55)	226 (55)	
Other, *n* (%)	138 (17)	65 (16)	73 (18)	
Vital sign at admission				
Heart rate, median (IQR)	108 (93–125)	109 (93–125)	107 (93–125)	0.694
Mean arterial pression, median (IQR)	54 (46–61)	56 (46–62)	54 (46–60)	0.205
Arterial blood gas at admission				
pH, median (IQR)	7.31 (7.21–7.38)	7.31 (7.21–7.37)	7.31 (7.22–7.38)	0.328
Lactate (mmol/L), median (IQR)	2.6 (1.5–5.1)	2.4 (1.4–4.8)	2.8 (1.5–5.5)	0.149
Therapy during first 2 days				
PCI, *n* (%)	131 (16)	65 (16)	66 (16)	0.500
CRRT, *n* (%)	173 (21)	91 (22)	82 (20)	0.247
IABP, *n* (%)	103 (13)	53 (13)	50 (12)	0.417
ECMO, *n* (%)	18 (2)	9 (2)	9 (2)	0.594
Impella, *n* (%)	35 (4)	16 (4)	19 (5)	0.365
TandemHeart, *n* (%)	12 (1)	7 (2)	5 (1)	0.386
Ventilator, *n* (%)	657 (80)	330 (81)	327 (80)	0.430
Therapeutic hypothermia, *n* (%)	7 (1)	3 (1)	4 (1)	0.500
Pacemaker, *n* (%)	66 (8)	33 (8)	33 (8)	0.551
Medications during first 2 days				
Epinephrine, *n* (%)	204 (25)	98 (24)	106 (26)	0.286
Norepinephrine, *n* (%)	339 (41)	171 (42)	168 (41)	0.444
Phenylephrine, *n* (%)	247 (30)	123 (30)	124 (30)	0.500
Vasopressin, *n* (%)	297 (36)	157 (38)	140 (34)	0.112
Dopamine, *n* (%)	167 (20)	80 (20)	87 (21)	0.301
Dobutamine, *n* (%)	221 (27)	113 (28)	108 (26)	0.376
Muscle relaxant, *n* (%)	93 (13)	51 (12)	42 (10)	0.189
⁣^∗^Max VIS, median (IQR)	18 (7–29)	17 (7–27)	18 (6–30)	0.332
SOFA score at admission, median (IQR)	8 (5–12)	9 (5–12)	8 (5–12)	0.499

Abbreviations: BMI, body mass index; CRRT, continuous renal replacement therapy; ECMO, extracorporeal membrane oxygenation; EN, enteral nutrition; IABP, intra-aortic balloon pump; IQR, interquartile range; PCI, percutaneous coronary intervention; SOFA, sequential organ failure assessment; VIS, vasoactive-inotropic score.

^#^: stroke include both of ischemic and hemorrhagic.

⁣^∗^MAX VIS is the maximum within 48 h of admission.

**Table 3:** Cumulative survival analysis, clinical outcomes, and complications for propensity score matching analysis.

	Early EN *N* = 409	Delay EN *N* = 409		95% CI	*p* value
Cumulative survival analysis			Hazard rate		
Mortality during 30 days			0.803	0.647–0.998	0.033
Mortality during 90 days			0.729	0.599–0.889	0.001
Mortality during 180 days			0.778	0.644–0.938	0.008
Clinical outcomes			Odds ratio		
Mortality at 30 days, *n* (%)	149 (36)	180 (44)	0.729	0.551–0.965	0.016
Mortality at 90 days, *n* (%)	175 (43)	226 (55)	0.606	0.459–0.798	< 0.001
Mortality at 180 days, *n* (%)	200 (49)	239 (58)	0.681	0.516–0.897	0.004
Complications			Odds ratio		
Ileus, *n* (%)	16 (4)	12 (3)	1.347	0.629–2.884	0.282
Aspiration pneumonia, *n* (%)	19 (5)	23 (6)	0.818	0.438–1.526	0.318
GIB, *n* (%)	41 (10)	32 (8)	1.313	0.809–2.130	0.163
Hospital stays (day), median (IQR)	12 (7–19)	17 (10–25)			< 0.001
ICU stays (day), median (IQR)	6 (4–11)	6 (4–11)			0.584

Abbreviations: CI, confidence interval; GIB, gastrointestinal bleeding; HR, hazard ratio; ICU, intensive care unit; IQR, interquartile range; OR, odds ratio.

## Data Availability

All raw data underlying the conclusions was extracted from MIMIC-IV database which is a conditional access database. Detailed database access conditions can be found at this website (ULR: https://physionet.org/content/mimiciv/2.2/). The code used to produce those results is extracted from MIT-LCP/mimic-code which is a public code in the GitHub (ULR: https://github.com/MIT-LCP/mimic-code).

## Supporting Information

Results of Adjusted proportional hazards model (COX) regression models.
