# Supplementary material for: Early Enteral Nutrition May Improve Survival in Patients With Cardiogenic Shock
Source: Emerg Med Int. 2025 Jan 6;2025:1465194. doi: 10.1155/emmi/1465194 (PMC11729513; doi:10.1155/emmi/1465194)
Supplement: Supporting Information — Additional supporting information can be found online in the Supporting Information section. [file 1465194.f1.docx]

**Supplementary Table 1.** Results of Adjusted proportional hazards model (COX) regression models

|  | **HR** | **95% CI** | **P-value** |
| --- | --- | --- | --- |
| **Adjusted proportional hazards model regression models for 30-day cumulative mortality** |  |  |  |
| EN plus prehospital characteristics^*^ | 0.821 | 0.667-0.995 | 0.042 |
| EN plus hospitalization characteristics^#^ | 0.781 | 0.627-0.972 | 0.027 |
| EN plus prehospital and hospitalization characteristics | 0.809 | 0.638-0.988 | 0.039 |
| **Adjusted proportional hazards model regression models for 90-day cumulative mortality** |  |  |  |
| EN plus prehospital characteristics^*^ | 0.737 | 0.604-0.898 | 0.002 |
| EN plus hospitalization characteristics^#^ | 0.704 | 0.577-0.859 | 0.001 |
| EN plus prehospital and hospitalization characteristics | 0.715 | 0.585-0.873 | 0.001 |
| **Adjusted proportional hazards model regression models for 180-day cumulative mortality** |  |  |  |
| EN plus prehospital characteristics^*^ | 0.785 | 0.650-0.948 | 0.012 |
| EN plus hospitalization characteristics^#^ | 0.746 | 0.617-0.902 | 0.002 |
| EN plus prehospital and hospitalization characteristics | 0.756 | 0.625-0.914 | 0.004 |

*Prehospital characteristics include age, gender, body mass index, all included comorbidities.

# Hospitalization characteristics include cause of CS, vital signs, arterial blood gas, medications, therapy, VIS and SOFA score.
